# The effects and determinants of exercise participation in first-episode psychosis: a qualitative study

**DOI:** 10.1186/s12888-016-0751-7

**Published:** 2016-02-20

**Authors:** Joseph Firth, Rebekah Carney, Lauren Jerome, Rebecca Elliott, Paul French, Alison R. Yung

**Affiliations:** Institute of Brain, Behaviour and Mental Health, University of Manchester, Room 3.306, Jean McFarlane Building, Oxford Road, Manchester, M13 9PL UK; Manchester Academic Health Sciences Centre, University of Manchester, Manchester, UK; Psychosis Research Unit, Greater Manchester West Mental Health NHS Foundation Trust, Manchester, UK; Department of Psychological Sciences, The University of Liverpool, Liverpool, UK

**Keywords:** Physical activity, Early intervention, Aerobic exercise, Resistance training, Recovery, Early psychosis, Community mental health, Rehabilitation

## Abstract

**Background:**

Previous qualitative studies have found that exercise may facilitate symptomatic and functional recovery in people with long-term schizophrenia. This study examined the perceived effects of exercise as experienced by people in the early stages of psychosis, and explored which aspects of an exercise intervention facilitated or hindered their engagement.

**Methods:**

Nineteen semi-structured interviews were conducted with early intervention service users who had participated in a 10-week exercise intervention. Interviews discussed people’s incentives and barriers to exercise, short- and long-term effects, and opinions on optimal interventions. A thematic analysis was applied to determine the prevailing themes.

**Results:**

The intervention was perceived as beneficial and engaging for participants. The main themes were (a) exercise alleviating psychiatric symptoms, (b) improved self-perceptions following exercise, and (c) factors determining exercise participation, with three respective sub-themes for each.

**Conclusions:**

Participants explained how exercise had improved their mental health, improved their confidence and given them a sense of achievement. Autonomy and social support were identified as critical factors for effectively engaging people with first-episode psychosis in moderate-to-vigorous exercise. Implementing such programs in early intervention services may lead to better physical health, symptom management and social functioning among service users.

**Trial registration:**

Current Controlled Trials ISRCTN09150095. Registered 10 December 2013.

**Electronic supplementary material:**

The online version of this article (doi:10.1186/s12888-016-0751-7) contains supplementary material, which is available to authorized users.

## Background

People with schizophrenia typically have poor physical fitness and lower levels of physical activity than general population [1, 2]. This is linked to the elevated rates of obesity, cardio-metabolic diseases and premature mortality observed within this patient group [3]. Furthermore, physical inactivity and poor fitness bears particularly strong relationships with negative symptoms and cognitive deficits in schizophrenia [1, 4], which strongly impair functional recovery and yet often remain untreated.

Exercise offers a possible adjunctive intervention which may improve both physical and mental health outcomes in schizophrenia. A recent systematic review and meta-analysis found that 90 min of moderate-to-vigorous activity per week can increase fitness, reduce positive and negative symptoms and improve cognition [5]. The qualitative literature has also shed light on how exercise may have these effects. Two separate reviews, each including between 11 and 13 qualitative studies, found that vigorous exercise can draw attention away from auditory hallucinations and/or adverse beliefs that people with long-term schizophrenia may have, and help them to ‘reconnect with reality’ by focusing on physical exertion [6, 7]. Furthermore, exercise may improve negative symptoms and real-world functioning through providing a valued, sociable activity with achievable and rewarding goals [6, 7].

Despite the clear benefits, adherence to exercise among people with schizophrenia is lower than other patient groups [8]. However, interventions which use supervised exercise have better rates of retention [8]. Additionally, interventions which accommodate individual preferences, through offering different types of exercise, have previously resulted in higher rates of exercise adherence among long-term schizophrenia patients [9, 10].

This is congruent with qualitative research which shows that patients feel that individualised support can overcome many of the barriers faced by people with schizophrenia, and facilitate exercise engagement [6, 7]. The qualitative literature has also provided insight into why people with schizophrenia often fail to achieve adequate amounts of exercise. For example, symptoms of paranoia and anxiety are barriers to attending exercise facilities, while amotivation and body-image issues can deter people from physically engaging [6].

However, all previous qualitative research has sampled patients who are receiving treatment for long-term schizophrenia. The experiences of exercise among younger patients within an ‘Early Intervention for Psychosis’ (EIP) service has yet to be explored. This is a pertinent area of enquiry, since early psychosis is a time when interventions which target negative and cognitive symptoms may maximize functional recovery [11]. It is also a ‘critical period’ for physical health interventions to prevent cardio-metabolic disorders from arising [12]. Patients themselves may feel more able to engage with exercise when they are at a younger age, with a lower BMI and in the absence of metabolic disorders [13].

Preliminary research has indicated that increasing physical activity and fitness during the first-episode of psychosis (FEP) can improve physical health and support functional recovery [14–16]. In a recent feasibility trial (the “iBeep” study) of an exercise intervention delivered through EIP services for patients with FEP, significant improvements were observed in cardio-metabolic health, positive and negative symptoms and cognitive functioning after just 10 weeks [17]. Additionally, adherence and retention rates were substantially higher than in previous exercise trials in schizophrenia [8, 17], perhaps due to the nature of the intervention applied, or certain characteristics of the first-episode sample.

We conducted a qualitative study of patients who had participated in the iBeep trial. The aim of this investigation was to explore the perceived benefits of exercise as experienced by people with FEP, and to establish the barriers and facilitating factors for increasing physical activity in this patient group. These findings could inform the development of future studies, and the implementation of exercise interventions within EIP services.

## Methods

### Ethics, consent and permissions

This study was conducted as the qualitative section of the iBeep (‘Investigating the Benefits of Exercise in Early Psychosis’) feasibility trial [17], which was approved by the North West Research Ethics Committee on 18/12/2013 (REC# 13/NW/0784) and registered with the current clinical trials database (ISRCTN: 09150095). Participants were recruited from EIP services in Greater Manchester, UK. In the United Kingdom, EIP services are currently offered to any individual aged 14–35 who are experiencing FEP, defined as full threshold psychotic symptoms for a period of greater than 7 consecutive days, regardless of formal diagnostic status. Additionally, diagnoses given during the early stages of illness are susceptible to change [18]. Therefore, no restrictions were placed on patients’ diagnosis in this study, in order to assess exercise as an intervention for FEP more broadly, and inform its implementation within EIP services. Inclusion criteria were: (1) currently receiving care for FEP as a service user of EIP services; (2) aged 18 – 35; (3) experiencing some psychological difficulties, defined as having either a score of ≥2 on the WHO Disability Assessment Schedule 2.0 [19] or ≥21 on the Beck Depression Inventory 2.0 [20]. Exclusion criteria were inability to provide informed consent, pregnancy, physical health issues which are a contraindication to exercise (as assessed by the referring clinician), and/or insufficient command of English to complete baseline assessments. All referrals were screened against inclusion criteria using a telephone interview. Eligible service users were then met in person to provide written informed consent.

### Intervention

Each participant received a 10-week individualised exercise training programme, which aimed to achieve ≥90 min of moderate-to-vigorous activity each week [17]. All interventions were delivered through ‘community leisure schemes’ within service users’ localities. Such schemes are now commonplace throughout the UK and offer highly discounted gym memberships and sports facilities to anyone referred by health services.

During the intervention period, participants were offered gym training sessions twice per week at their local leisure centres. These were supervised by research assistants, either on a 1-to-1 basis or in small groups of 2–3 participants. Research assistants had no formal exercise qualifications but did have several years of exercise experience. Their role was to facilitate exercise adherence through arranging gym memberships, accompanying participants to gym sessions and recording their training. A standardised training plan was developed in line with recommendations from exercise physiologist JM, and used as a guide for exercise sessions, consisting of combination of aerobic and resistance exercise activities (See Additional file 1; Aerobic-resistance training plan for IBEEP). However, the specific content of each session was tailored toward participant preference. Mandatory gym inductions were also provided by the personal trainers at community leisure centres (who were available for further support throughout the duration of the intervention).

### Qualitative interviews

Informants for the qualitative section were selected on the basis of availability while aiming to obtain a “maximum variation sample” [21] of men and women, older and younger participants, full compliers and non-adherers. Participants were interviewed on a one-to-one basis. Interviews took place either immediately after the intervention, or 6-months after the supervised training period had subsided. These two time-points were used in order to obtain additional perspectives from participants following the withdrawal of supervised training, thus providing a more complete understanding of exercise adherence, benefits, and barriers experienced by patients over time.

All interviews used topic guides which contained open ended questions about participant experiences and opinions of exercise, and were conducted between June 2014 and March 2015. To minimize response bias, interviews were conducted by different research assistants to those who supervised the exercise sessions. However, due to unavailability of research assistants at follow-up, some interviews were conducted by the same person who had previously supervised participants’ exercise. Nonetheless, this only occurred for five of 19 total interviews, each of which was conducted 6 months after the intervention had finished, thus limiting potential impact on findings.

### Data analysis

The current study pre-specified several areas of interest for analysis, determined on basis of existing qualitative literature around exercise and schizophrenia [6, 7]. These areas included; possible reasons for exercising; short- and long-term effects of exercise; barriers/facilitating factors towards exercise participation. A thematic analysis was used to determine the key themes from participants’ dialogue on these topics, through applying the five stages of qualitative analysis [22]:

(i) transcripts were read and re-read to familiarize the researcher with the data
(ii) a range of codes were generated to index common features across interviewees
(iii) these initial codes were examined to determine prevalent themes
(iv) themes were reviewed for internal homogeneity and external heterogeneity, to combine similar themes into overarching themes and draw coherent links or distinctions between them [21]
(v) the overarching themes were firmly defined, organized in relation to their collated data extracts, and then analysed for sub-themes.

### Interpretive rigor

To reduce the risk of bias, the prominence of overarching themes and their respective sub-themes was determined through discussion between three of the authors, who had transcribed the interviews and reviewed all coded quotations for each theme. To validate the findings, first hand quotes are presented word-for-word within the Results and Table 1, following removal of non-specific interjections [23].

**Table 1:** Baseline characteristics of participants

Characteristic	Interviewees (*n* = 13)	Total sample (*n* = 31)
Gender		
Male; n (%)	12 (92)	25 (81)
Female; n (%)	1 (8)	6 (19)
Age, years; mean (s.d.)	26.5 (4.5)	25.8 (4.6)
Time in EIS, years; mean (s.d.)	2.0 (1.3)	1.9 (1.4)
ICD-10 Diagnosis; n (%)		
Non organic psychosis	6 (46)	15 (48)
Schizophrenia	5 (38)	9 (29)
Schizoaffective disorder	1 (8)	3 (10)
Bipolar disorder	0 (0)	1 (3)
Other psychotic disorder	1 (8)	3 (10)
Physical and Mental Health		
BMI, kg/m2: mean (s.d.)	32.4 (7.2)	30.4 (6.9)
PANSS total; mean (s.d.)	76.7 (12.8)	79.0 (18.0)
PANSS positive; mean (s.d.)	19.8 (5.7)	18.9 (6.2)
PANSS negative; mean (s.d.)	17.5 (3.0)	19.0 (6.1)
BDI-II total; mean (s.d.)	25.1 (12.7)	21.7 (10.9)
SOFAS; mean (s.d.)	47.5 (9.0)	46.6 (8.0)

*BDI-II* beck depression inventory, *BMI* body mass index, *EIS* early intervention services, *ICD-10* International Classification of Diseases 10th Edition, *PANSS* positive and negative syndrome scale, *SOFAS* social and occupational functioning assessment scale

## Results

The clinical and demographic characteristics of participants are displayed in Table 1, which shows that interviewees were broadly representative of the entire ‘iBeep’ sample. A total of 19 interviews were conducted; nine immediately after the 10-week intervention and a further ten at 6-month follow-up. Six participants were interviewed at both time points. The mean level of exercise achieved by these participants during the intervention was 119 min of moderate-to-vigorous exercise per-week for 10 weeks (s.d. 74 mins), primarily in the form of individualised gym training. Three overarching themes emerged from the thematic analysis. These were: (a) exercise alleviating psychiatric symptoms (b) improved self-perceptions from exercise, and (c) factors determining exercise participation. To accurately represent the entirety of participants’ dialogue, one exemplar quote for each theme has been featured in Results (selected through mutual agreement between authors), with three additional examples in Table 2.

**Table 2:** Additional examples of content within each qualitative sub-theme

Theme (and subthemes)	Additional example quotes
(A) Exercise alleviating psychiatric symptoms	
(A1) Reducing positive symptoms	P011: “It’s like, when I’m doing the weights or I’m running, I don’t sit there and think about the voices or what they’re saying, I just think ‘yeah let’s do it!”
	P006: “All your mental issues are going to go away because once you’re concentrating on picking up weights and doing exercise. Then your mind goes somewhere else.”
	P008: “It makes you calmer when exercising. It gives you a fresh mind. If you have got any worries then just it’ll go away.”
(A2) Overcoming negative symptoms and depression	P012: “I felt the change, emotional changes, you know what I mean, that I was like flooded with chemicals if you will, like made me feel good by myself”
	P007: “I think more energetic a bit more enthusiastic kind of thing. Yeah more up for doing things which I probably normally wouldn’t do”
	P004: “More energetic, feeling happier, makes you feel a lot happier I think. Erm you feel motivated to do things - and energetic.”
(A3) Supporting psychological well-being	P006: “Go and exercise you’ll feel you’ll feel your minds gone free. And you’re stress free. You’ll get all your worries to go away, cos you’re concentrating on something else”
	P005: “A lot of people don’t understand how much exercise helps. But if I didn’t do exercise I’d be in a lot worse place than what I am right now cos at times when I feel like I’m just so angry, and stuff like that, I’ve gone training, I’ve done press ups and stuff at home and it’s cleared my head, killed me anger and you feel better for it…”
	P003: “Yeah, makes you happier really. I don’t know really, just when you’ve done it you’re buzzing aren’t you.”
(B) Improved self-perceptions from exercise	
(B1) Overall confidence	P002: “Has a big err, a big big er, has a big effect on all of it. It makes me feel more happy in myself, you know, more confident, more self erm… [esteem], you know, believe in myself better, a lot more.”
	P012: “once you once you go gym and that after a while you start feeling good about yourself. You start walking round like yeah I’m getting big and do you know what I mean type thing.”
	P011: “I love it. It’s just like, thinking yeah, I couldn’t do this 4 months ago, now look at me doing it comfortably. Let’s throw it up a gear!”
(B2) Benefits extending to other areas of life	P001: “I feel good knowing that I’ve lost weight. I’m really excited and proud of meself that I’ve lost weight…Erm I go out with my friends more and they are like proud of me because they know I’ve lost weight because they know about me wanting to lose it and they have been very supportive as well.”
	P005: “it’s a lot better when you start doing it and like I said you gain that confidence up and you think you just think to yourself I can do this. I’m training and, er give me another year year of doing this I think I’ll be ready to go back to work…and you’ll be nervous getting a job interview or anything, like anyone would, but you’ll go in with your confidence that you gained from going from doing your exercise. And when er like people from interviews see that, you work hard in the gym or something like that they’ll look at you and they’ll think well this guy’s got quite good confidence.”
	P009: “My head felt a bit better after I’d done the exercise, like it was easier to do uni work and…. I dunno my head just felt a lot clearer.”
(B3) Sense of achievement	P002:” Yeah I feel like I’ve achieved summat I feel like you know a lot better in myself I feel like, I should do this every day”
	P005: And you think yeah I feel good, and erm especially if you got somebody with you you know you’ll have it all out that were a good session that and then you’ll go you’ll sit down or you go home you’ll turn your xbox on or you do your house cleaning or something like that and then when you’re going to bed you think I had a good day today
	P012: “For me…it’s not so much as like putting weight on or losing weight or it was just that feel good factor that I enjoyed…I used to look forward to going to the gym for that particular reason you know to make me feel good and that.”
(C) Factors determining participation	
(C1) Acceptability of individualised routines	P003: “It was good that we got to choose something out of like a list. We could have done all different things… I thought the options were quite good. But I would have like to have done boxercise as well.”
	P007: yeah let them try all the different er materials out there in the gym um different weights machines, definitely they will fit into something that they like yeah
	P004: “I think it’s er finding what they enjoy what they love to do… And if you can maybe, er, assess what type of exercise would be desirable um then, er, I’m sure they they’re more likely to exercise”
(C2) Importance of a training partner	P006: “It wasn’t just him [the trainer] standing and shouting at you. He was like persuading us on like ‘come on you can do it, you can do it!’ when he says things like that it makes me more activate.”
	P005: “[You’ve] got someone there also who’s basically saying, ‘it’s alright I’m here. I’ll show you what you need to learn’ and once like the 6 months is over they’ve got that extra bit of confidence to say I’ll give it a go on me own.”
	P010: “Yeah definitely I think group work group exercising gets you more motivated than…Whereas if you’re going on your own you don’t really motivate yourself especially this time of year as well.”
(C3) Overcoming anxiety and motivational barriers	P001: “…if you go on your own you like don’t know somebody and you feel dead shy and timid and stuff like that, whereas if you go with somebody you’re chatting as you’re swimming or on bikes or at gym or something the time passes and stuff.”
	P002: “They push you to do more than you would and if you’re on your own you might just say oh well I’m not really bothered, but if someone’s with you they’ll they’ll push you to do more they’ll push you to go.”
	P004: “What really gave me the confidence to go, because the err, we had to work in sort of a group, so we worked in a group and did some sets and stuff, so that was really good. It made me feel a lot secure.”

(A) **Exercise alleviating psychiatric symptoms**
  Participants generally felt that exercise was capable of providing acute relief for psychiatric symptoms. These effects varied across participants. Some reported relief from positive symptoms (i.e. auditory hallucinations and paranoia) and others for negative symptoms (amotivation and anhedonia). This seemed to depend on which aspects of psychosis characterised the individual’s current condition.
  1. *Positive symptoms*
    Participants described how the physical demands of exercise temporarily subdued positive symptoms, by helping them to direct their thoughts away from intrusive voices, delusions and/or paranoia:
    P005: “Well with psychosis, which is partly what I suffer from, I hear the voices and stuff like that. But when I’m training, the voices are suppressed, cos I’m so concentrated on lifting that weight up, doing the next exercise, that everything else clears out of your head.”
  2. *Overcoming negative symptoms and depression*
    Exercising was also beneficial for negative symptoms and depression, reportedly helping people to overcome feelings of low energy, low mood and amotivation.
    P006: “it makes me more active, before I was lazy and couldn’t be bothered doing things. But now it’s like I’m more active and I just want to go out there and start going to the gym… Got my get up and go back!”
    Furthermore, comments about this were often tied to feelings of exercise acting directly on one’s mood state through stimulating the production of neurological chemicals or ‘endorphins’:
    P002: “I always get them bad days but when I go the gym, like I said, physical exercise, it lets endorphins off in your brain doesn’t it. So even if you are a bit depressed it can erm make you not depressed, [laughs] or make you slightly more happy.”
  3. *Supporting psychological well-being*
    Participants explained how these immediate effects of exercise provided a sense of well-being and freedom from mental health problems. This could have an enduring impact on the rest of one’s day, reducing usual feelings of disturbance, stress and/or despair.
    P009: “…I’d notice, after I’d done the gym sessions, I felt really fresh. Like afterwards like my mind felt kind of washed, if you like.”
(B) **Improved self-perceptions following exercise**
  1. *Overall confidence*
    While engaging in exercise was associated with transitory relief from mental health issues, the long-term benefits of exercise participation were more strongly related to improved self-perceptions. This was described as an increased confidence, stemming from greater self-efficacy and self-esteem.
    P003: “I think people can benefit from it - to get fitter and healthier - Get more confidence and self-esteem.”
  2. *Benefits extending to other areas of life*
    Improvements in confidence were often directly linked to physical changes, in terms of feeling fitter, healthier and/or obtaining a better body image. Others described exercise as providing a supportive platform for improving social confidence. In both cases, the increased confidence gained from exercising positively affected other areas.
    P007: “if you can learn and do things in the gym, then you can do it anywhere outside and, you know, you can get other people involved as well. And er it definitely helps your lifestyle you know - it improves your lifestyle.”
  3. *Sense of achievement*
    People also received an immediate boost from a ‘sense of achievement’ associated with completing exercise sessions. This was a highly prevalent experience, reported by almost all.
    P010: “Just lifts your spirits I think when you’ve done it and just makes you feel like you’ve achieved a goal in a day when you go to the gym and if you go swimming or…Definitely lifts it yeah, bit of exercise does.”
(C) **Factors determining exercise participation**
  1. *Acceptability of individualised exercise training*
    Overall, participants’ achieved more than satisfactory amounts of exercise during their 10-week programmes. We found that the exercise offered (most often personalised gym training) was acceptable and desirable among people with first-episode psychosis. From the interviews, it was apparent that flexible, individualised interventions ensure that exercise is enjoyed, and thus adhered to by participants.
    P004: “…it’s very important in drawing the individual towards making their own choice, because it is about choice. They can make a choice in what they want to do, and what they love to do, it motivates them to sort of do what they feel happy to do.”
  2. *Importance of a training partner*
    Another emergent sub-theme was the perception of exercise as an inherently social activity. A central aspect of the ‘iBeep’ intervention was the provision of a research assistant to facilitate exercise on either an individual or small-groups basis (2–3 participants). This was found to be highly effective, perhaps essential, for encouraging attendance and promoting engagement with exercise.
    The only recurrent criticism of ‘iBeep’ was the relatively short length of the intervention (10 weeks), with participants unanimously stating that a longer (or permanent) period of supervised exercise sessions would have been preferred. Furthermore, participants’ suggestions for further and/or alternative exercise interventions all involved some level of social support, such as; personal trainers, ‘service user friendly’ exercise groups, or learning how to train with friends.
    P001: “It’s just summat’ I want to do and it’s just a bit of support and more encouragement to do it. And I got the encouragement and the support, so I did it.
  3. *Barriers towards exercise*
    Barriers towards exercise fell into two categories. The most frequently mentioned was anxiety within the exercise environment. This was often related to social anxiety, or a lack of exercise efficacy. The second was feelings of low motivation, which dissuaded some participants from initiating exercise alone. However, this was never described as apathy towards exercise itself, as they still perceived exercise as beneficial and congruent with personal goals. Instead, participants expressed how generalized amotivation could become a psychological obstacle towards initiating any activity, even those which they valued and enjoyed. Nevertheless, the feedback showed that having an experienced and enthusiastic trainer can overcome both amotivation and anxiety:
    P012: “It’s just good to have a partner you know mentally it’s good having a partner. You know what I mean; when you go out there on your own you just feel like, you know, like you really need somebody to come with you I suppose.”

## Discussion

This study examined the experiences of exercise among people with first-episode psychosis who had recently participated in a 10-week intervention. A thematic analysis showed exercise could provide relief for participants’ positive symptoms along with improving their energy levels and mood. Social aspects of exercise also emerged as overarching themes, in terms of (i) long-term benefits of exercise participation, and (ii) ideal types of support required. The findings are consistent with qualitative studies of exercise in people with long-term schizophrenia [6, 7]. They are also congruent with quantitative data from the ‘iBeep’ study, which found significant reductions in participants’ positive and negative symptoms after the exercise intervention, as measured by the PANSS [17].

### Exercise as an intervention for first-episode psychosis

The positive symptoms of psychosis can be indications of a ‘loss of contact with reality’ [24]. People with long-term schizophrenia have previously described how exercise provides a platform to reconnect with a reality, thus helping to detach from these symptoms [25–27]. Our study replicated these findings in the first-episode sample; participants similarly described how exercise can inhibit auditory hallucinations and intrusive thoughts, as a consequence of physical exertion demanding their full attention. Exercise was also perceived as energizing and uplifting, making participants feel more enthusiastic about other aspects of life. This may be linked to physiological responses to exercise, as participants reported a rush or ‘feel good’ effect immediately after their sessions. Therefore, exercise offer a destigmatising, empowering and natural method for overcoming negative symptoms in early psychosis, which are usually unresponsive to antipsychotic treatment [28].

As the benefits of exercise for psychiatric symptoms were often tied to the physical activity itself, rather than just the social aspects, it is important to provide activities which allow for sufficient levels of exercise-intensity. While low-intensity activities (e.g. walking, stretching) may have other therapeutic benefits, moderate-to-vigorous exercise may be optimal for people with psychosis, as it would occupy enough attentional resources to subdue voices/delusions, while also physiologically stimulating the body to release neurobiological chemicals known to enhance mood [29]. This is supported by a recent meta-analysis, which found that the symptoms of schizophrenia are only reduced significantly by exercise interventions which implement at least moderately intense exercise [5]. Future randomised trials should aim to specify the mechanisms of exercise as an intervention for FEP using time-and-attention control conditions, in order to separate the physiological effects of vigorous physical activity from the social support provided during the intervention.

Moderately difficult or ‘challenging’ exercise has the added benefit of providing an immediate sense of achievement. Participants experienced this upon completing their sessions, and described how this led to improved self-perceptions overtime. For instance, many participants explained how achieving their goals in the exercise setting had increased their self-efficacy and confidence for other areas of life, which may help to improve psychosocial functioning.

### Overcoming barriers using individualised exercise training

Participants discussed how sufficient levels of exercise can be attained by tailoring programmes to individual needs, thus facilitating physical exertion through intrinsically rewarding activities. Early psychosis may be an ideal time to implement this, when patients are younger, fitter, and less likely to have cardio-metabolic diseases, which may act as a barrier towards exercise [13, 30]. Among our participants, barriers were mostly related to anxiety, rather than the physical health complaints often mentioned by long-term patients [30].

Additionally, motivational barriers were not attributed to reduced interest in exercise, but rather as generalized amotivation obstructing their engagement with positive activities. Crucially, these barriers were readily overcome through the intervention, with participants achieving 107 min of moderate-to-vigorous activity per week on average [17]. Thus, both the qualitative and quantitative data shows individualised exercise interventions can feasibly achieve sufficient moderate-to-vigorous activity for people with early psychosis.

Providing structured advice is insufficient for overcoming barriers towards exercise [31]. Instead, social support was consistently identified as the crucial facilitator of exercise engagement – as is found in long-term schizophrenia [32–34]. Participants described how the provision of a ‘training partner’ moved them from feeling willing but unable to exercise, to readily achieving substantial amounts of activity each week. This is likely because a training partner can effectively address both of the most prominent barriers towards exercise (anxiety and amotivation), through providing reassurance or prompting as required.

Research assistants who supervised exercise in this study were not qualified personal trainers, but were familiar with gym-based training. This suggests that such interventions could be delivered by any mental health support staff with personal exercise experience. Additional exercise support was available from the qualified exercise professionals based in community leisure centres. Thus future interventions could also take advantage of the expertise and facilities readily available through community facilities, to provide low cost and destigmatising interventions to this population.

Furthermore, due to the general alignment in exercise preferences expressed by participants [35], research assistants were often able to group participants and supervise 2–3 people per session. Administering individualised exercise within small group settings has also proved feasible in studies of long-term schizophrenia patients [9, 10]. Thus, implementing this approach within clinical practice would reduce the cost of exercise interventions, and may even improve long-term maintenance of exercise through enabling participants to act as each other’s training partner after the supervised period subsides.

### Limitations

One limitation is that our sample were mostly male (92 %) and had demonstrated an existing desire for exercise by opting into the study. Thus, findings may not generalise across whole first-episode population. However, the high referral-to-recruitment rates [17], along with the many benefits observed, suggest that a substantial portion of early intervention service users would value exercise as an adjunctive intervention. It should also be considered that the individualised nature of our intervention meant that each participant’s preferences were flexibly accommodated for. Therefore, barriers which may typically prevent people from engaging (i.e. lack of interest in activities offered, inaccessibility of facilities) would be less likely to arise.

For this study, we conducted one round of interviews immediately following the intervention, and then another 6-month after, in order to assess patient opinions on exercise after the supervised training period had subsided. A limitation here is that, in order to obtain sufficient data, six of the original interviewees were also used in follow-up interviews, resulting in more qualitative data being available for the subgroup (*n* = 6) who were interviewed at both time-points. Nonetheless, the data was grouped by participant number throughout, and thematic analysis was applied to identify the common themes across interviewees (rather than just interviews) [21, 22], thus reducing the possibility of ‘double counting’. Furthermore, each emergent sub-theme was supported by quotations from multiple participants.

## Conclusions

The findings show that interventions which allocate support on an individual-needs basis, while also prioritising the amount/intensity of exercise (rather than the modality), are well-suited for people with early psychosis. Such interventions could be used in EIP services to provide patients with an additional tool to manage positive and/or negative symptoms, while also improving physical health and psychosocial functioning. Future research must establish sustainable systems for implementing exercise in clinical practice. Optimal systems could incorporate short-term ‘introductory periods’ with relatively high levels of supervision, followed by step-down periods or peer support, leading people to autonomy and social integration with their own exercise routines at local facilities.

## Additional file

Additional file 1:
**Gym Training Guide used for IBEEP**. (PDF 355 kb)
