# Supplementary material for: The effects and determinants of exercise participation in first-episode psychosis: a qualitative study
Source: BMC Psychiatry. 2016 Feb 20;16:36. doi: 10.1186/s12888-016-0751-7 (PMC4761413; doi:10.1186/s12888-016-0751-7)
Supplement: Additional file 1: — Gym Training Guide used for IBEEP. (PDF 355 kb) [file 12888_2016_751_MOESM1_ESM.pdf]

iBeep

Session 1

15 mins - *Cardio Training:* Bike or Treadmill or Rowing machine

30 mins - *Weight Training:* Do 3 sets of 12 reps for each exercise

1) Bench Press or Machine

3 X 12

2) Pull down machine

3 X 12

3) Squats or Leg Press

3 X 12

4) Shoulder Press or Machine

3 X 12

5) Bicep Curls

3 X 12

6) Core Training

3 X 12

15 mins - *Cardio Training:* Bike or Treadmill or Rowing machine

Session 2

15 mins - *Cardio Training:* Bike or Treadmill or Rowing machine

30 mins - *Weight Training:* Do 3 sets of 12 reps for each exercise

1) Seated Row Machine

3 X 12

2) Dumbbell Press or Machine

3 X 12

3) Upright Rows (bar or cable)

3 X 12

4) Bicep curls (bar or cable)

3 X 12

5) Triceps Pushdowns

3 X 12

6) Core Training

3 X 12

15 mins - *Cardio Training:* Bike or Treadmill or Rowing machine
